# Neurodiversity as an epistemic stress test for psychotherapy and health care: a neuroaffirmative conceptual analysis of relational-proximity priors in clinical, institutional, and AI-supported models

**DOI:** 10.3389/fpsyt.2026.1862968

**Published:** 2026-09-10

**Authors:** Eik Niederlohmann

**Affiliations:** Department of Psychosomatic Medicine and Psychotherapy, Kliniken Erlabrunn, Breitenbrunn, Germany

**Keywords:** double empathy problem, epistemic injustice, human-centered AI, neuroaffirmative care, neurodiversity, psychotherapy, public mental health, relational-proximity priors

## Abstract

Neurodiversity-affirming mental health care requires not only adapting support to neurodivergent people but also examining the relational assumptions through which alliance, empathy, cooperation, progress, and risk are interpreted. This Conceptual Analysis develops the heuristic concept of relational-proximity priors: implicit assumptions that emotional closeness, rapid reciprocity, eye contact, visible warmth, and ready acceptance of help are generally reliable indicators of therapeutic fit or improvement. Drawing primarily on autism research and, more cautiously, on broader neurodivergence-informed literature, the paper synthesizes work on neurodiversity, double empathy, minority stress, healthcare accessibility, interpersonal distance, epistemic injustice, and psychotherapy process. The argument is that when such priors remain unaudited, clinically meaningful behaviors such as distance, reduced affect display, written communication, slower pacing, or literal style may be misread as resistance, poor alliance, or lack of insight. The paper further proposes that these priors can become sedimented in service routines, documentation practices, and AI-supported systems if observation and interpretation are not adequately separated. The article does not present a validated empirical construct or a systematic review; rather, it offers a theory-anchored conceptual synthesis and a set of heuristic audit tools intended to slow interpretation and widen clinically plausible hypotheses. Implications are discussed for psychotherapy, service design, documentation, participatory evaluation, and AI governance in public mental health.

## 1 Introduction

Neurodiversity has become a key site for reconsidering what counts as mental health, good care, and human flourishing. In contemporary neurodiversity scholarship, distress is not understood only as an intrapersonal property or as the simple outcome of a disordered nervous system; it is also shaped by minority stress, environmental fit, epistemic exclusion, and the design of services themselves (1–6). For Public Mental Health, this shift matters because it relocates part of the problem from the individual to the interaction between persons, institutions, and social norms.

This relocation is especially important in psychotherapy and health care. Many clinical encounters depend on unspoken assumptions about what a good therapeutic relationship should look like: emotionally warm, visibly reciprocal, verbally fluent, comfortable with eye contact, grateful for help, and progressively more open. These expectations may be clinically useful in some encounters, but several strands of research suggest that they can become unreliable when clinicians work with autistic people and, more cautiously, with some other neurodivergent people whose communication, sensory regulation, pacing, or help-seeking differs from majority-coded norms (9–14).

This paper argues that neurodiversity can be understood as an epistemic stress test for such models of care. A stress test does not presuppose that one group is globally more correct than another. Rather, it reveals where a system fails when its hidden assumptions are exposed. The proposed hidden assumptions are termed relational-proximity priors: background expectations about how much closeness, warmth, reciprocity, affective display, spontaneity, eye contact, and visible acceptance of help should be present for an interaction to count as empathic, collaborative, or therapeutic.

When relational-proximity priors are treated as universal rather than situated, autistic forms of distance, literalness, low-intensity reciprocity, asynchronous communication, or explicit boundary-setting can be misclassified as pathology, resistance, poor alliance, or lack of motivation (13–20). The broader neurodivergent relevance of this claim is plausible but not settled. It requires profile-specific calibration, especially because ADHD, dyslexia, Tourette syndrome, intellectual disability, acquired neurodivergence, and other forms of neurocognitive difference do not share one relational phenotype.

The paper develops a neuroaffirmative conceptual analysis with four linked aims: to define relational-proximity priors more precisely; to specify the method and limits of the conceptual synthesis; to show how autistic and neurodivergence-informed perspectives stress-test clinical interpretation, institutional routines, and AI-supported models; and to derive practical safeguards for psychotherapy, service design, documentation, and public mental health governance. The practical claim is modest but consequential: a care system cannot call itself neuroaffirmative if it only asks how neurodivergent people can move toward neurotypical proximity, but not how care itself can move toward forms of distance, explicitness, pacing, and structure within which people can remain safe, agentic, and real.

## 2 Methodological approach and literature basis

This article is a Conceptual Analysis using a structured, theory-driven narrative and conceptual approach. It does not present original empirical data, a systematic review, or personal clinical material. It is informed by methodological literature on concept analysis and concept development, especially the distinction between concept analysis as a strategic examination of the state of the literature and concept advancement as refinement of a concept for future inquiry (7, 8). The aim is therefore not to validate relational-proximity priors as a measurable construct, but to define a clinically recognizable, theory-anchored heuristic and to specify the conditions under which it should be tested empirically.

The conceptual corpus was delimited around five literature domains whose intersection remains clinically under-theorized: neurodiversity and neuroaffirmative scholarship; research on double empathy, neurotype matching, interpersonal distance, and autistic peer interaction; public mental health literature on minority stress, masking, healthcare accessibility, and quality of life; interpersonal, psychodynamic, and ethical work on empathy, relational safety, countertransference, and epistemic injustice; and debates on trustworthy AI, clinical documentation, and health-care annotation (1, 5, 9, 13, 15, 19, 21–26). Sources were selected because they helped clarify at least one of the following: relational mismatch, service-access barriers, interpretive risk, communicative fit, boundary conditions, or the translation of clinical labels into institutional or computational systems.

**Figure 1:**
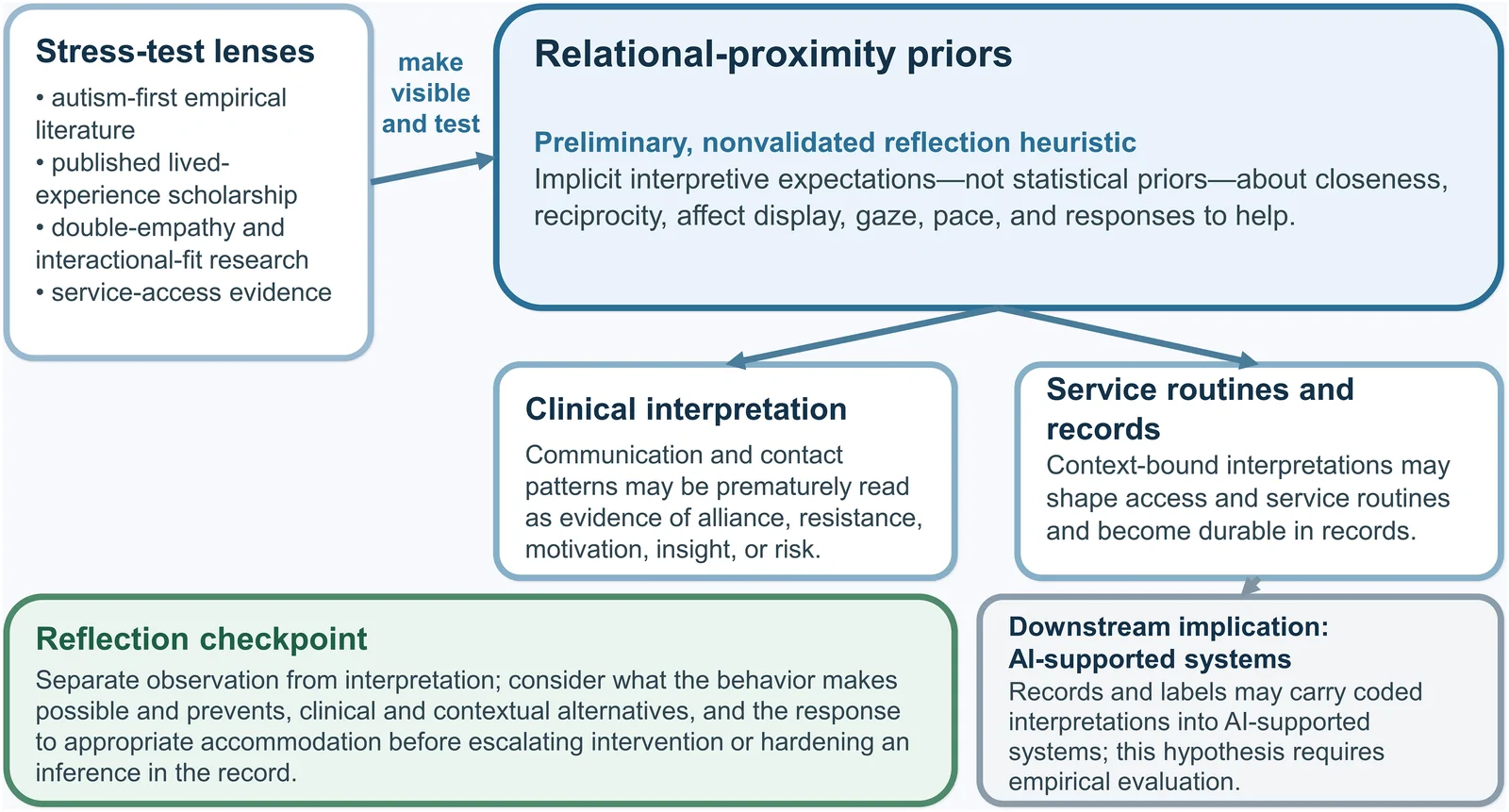
Conceptual map of relational-proximity priors. Autism-first empirical literature, published lived-experience scholarship, double-empathy and interactional-fit research, and service-access evidence serve as stress-test lenses for implicit relational expectations. These expectations may shape clinical interpretation, service routines, and records; records and labels may carry relationally coded interpretations into AI-supported systems. This downstream governance hypothesis requires empirical evaluation. The schematic is a preliminary, nonvalidated reflection heuristic, not an assessment, certification, or formal audit tool. Arrows denote proposed conceptual relationships, not validated causal effects. Transfer beyond autism requires profile-, person-, and context-specific study; neither distance nor closeness has a fixed meaning, and clinical and contextual alternatives remain necessary.

The analytic procedure had six steps. First, the target problem was defined as the recurring misinterpretation of distance, reduced affect display, low-intensity interaction, written communication, or delayed reciprocity as poor alliance, resistance, noncooperation, or lack of insight. Second, candidate attributes were extracted across the literature: closeness, warmth, reciprocity, gaze, pacing, help acceptance, gratitude, and affective legibility. Third, contrasts and boundary cases were examined, including situations in which distance is protective accommodation, defensive avoidance, depressive withdrawal, psychotic disconnection, trauma response, or sensory regulation. Fourth, the same attributes were compared across three levels: clinical encounter, institutional routines, and AI-supported documentation or annotation. Fifth, the proposed construct was refined to focus on background interpretive expectations rather than on neurodivergence, proximity, or empathy as such. Sixth, **Tables 1**–**3** were derived as heuristic audit tools that slow interpretation and make hidden priors explicit. The conceptual pathway is summarized in **Figure 1**, which shows how the stress-test lenses make relational-proximity priors visible and how such priors may shape clinical interpretation, service routines and records, and downstream AI-supported systems.

**Table 1:** Hidden relational-proximity priors and neurodiversity stress-test questions.

Hidden relational-proximity prior	Common misattribution if unexamined	Profile-calibrated neuroaffirmative stress-test question
More closeness means better alliance	Distance is read as poor alliance, withdrawal, or lack of trust.	Could predictability, lower social demand, side-by-side contact, or written exchange be the form in which alliance becomes possible here?
Emotional warmth is usually empathic	Warmth is intensified even when it feels invasive, ambiguous, or overwhelming.	How is this person likely to experience my tone, pacing, facial affect, and emotional intensity in this moment?
Eye contact signals engagement	Reduced eye contact is read as disinterest, avoidance, or pathology.	Is gaze reduction functioning as sensory regulation, concentration, or protection rather than disengagement?
Visible acceptance of help signals readiness	Requests for space, time, writing, or fewer people are read as rejecting care.	What form of help is acceptable without loss of autonomy, overload, or increased masking pressure?
Spontaneous reciprocity means progress	Masking, appeasing, or social smoothing is mistaken for improvement.	Am I rewarding neurotypical performance rather than authentic participation, safety, and self-determination?
Low affect means low connection	Minimal facial or vocal signaling is read as emotional absence or lack of depth.	Could low-intensity expression be a stable communication style or regulation strategy rather than lack of feeling?
Regulation that reduces proximity is resistance	Distance, structure, limited affect display, or written communication are interpreted as defensive avoidance.	What does this strategy make possible, what does it prevent, and what does it cost over time? Has the service adapted sensory, communicative, and autonomy demands before interpreting the behavior as defensive?
Staff discomfort indicates patient pathology	Countertransference is projected outward as a patient problem.	What expectation of reciprocity, gratitude, speed, or reassurance in me or the team may be frustrated right now?
Normalized social fit is the goal of care	Outcome is defined as looking less neurodivergent or being easier for the service to read.	Would this change still count as progress if achieved without masking, exhaustion, appeasement, or loss of self?
One neurodivergent profile generalizes to all	Autism-informed safeguards are applied as if they fit every neurodivergent person.	Which profile-specific, person-specific, cultural, and situational factors should calibrate this question?

**Table 1** is a theory-anchored heuristic audit aid derived from the conceptual synthesis; it is not a validated measurement instrument and should not replace clinical formulation.

**Table 2:** Professional countertransference risks and neuroaffirmative alternative responses.

Clinical or team-level challenge	Typical escalatory response	Neuroaffirmative alternative response
The patient responds briefly and without visible warmth.	Increase relational pressure, pursue disclosure, or read the patient as rejecting help.	Lower the intensity of contact, make expectations explicit, and ask which form of exchange is easiest to use right now.
The patient declines eye contact or prefers side-by-side/written communication.	Treat the preference as resistance or poor social relatedness.	Reframe the preference as possible regulation; adapt the medium before interpreting the motive.
The clinician feels ineffective, unseen, or unappreciated.	Rescue harder, over-explain, over-offer, or moralize gratitude.	Use supervision to examine wishes to feel helpful, mirrored, or effective without blaming the patient.
The team becomes anxious about limited reciprocity or slow trust-building.	Converge on narratives of poor alliance, noncompliance, or lack of readiness.	Ask what proximity norm the team is using and whether the patient is being required to over-adapt to the service.
The patient appears more socially smooth over time.	Assume that improvement is self-evident.	Assess whether the change reflects safety and agency or costly masking, appeasement, and exhaustion.
Distance is experienced as a rupture.	Intensify warmth or interpretation in order to repair quickly.	Consider whether distance is a prerequisite for continued contact and whether repair requires less, not more, proximity.
Literal, monotone, or highly focused communication feels hard to read.	Infer lack of insight, mentalization, or emotional depth.	Separate observation from interpretation and check meaning over time, across contexts, and across modalities.

**Table 2** frames countertransference risks as supervision questions, not as evidence that any particular patient behavior has one fixed meaning.

**Table 3:** Relational-proximity audit across clinical, institutional, documentation, and AI-supported models.

Level of analysis	What gets overfit to majority-coded norms	Audit questions/neuroaffirmative safeguards
Clinical encounter	Alliance, motivation, insight, empathy, and risk are inferred from warmth, eye contact, verbal ease, and rapid reciprocity.	Distinguish observation from interpretation; ask what dosage and form of contact the person can use; treat distance as possible regulation before treating it as resistance.
Supervision/team process	Staff comfort and felt helpfulness become implicit markers of treatment quality.	Use supervision to surface countertransference, rescue dynamics, and unspoken proximity ideals; review whether the patient is carrying most of the adaptation burden.
Service design	Telephone access, noisy settings, groups, unstructured interviews, and fast transitions are treated as ordinary and neutral.	Offer multiple communication channels; reduce sensory burden; increase predictability; make pacing, boundaries, and accommodations explicit.
Documentation and records	Labels such as poor alliance, guarded, flat affect, or noncompliant harden context-bound interpretation into institutional truth.	Document context, sensory load, communication preference, masking concerns, accommodation attempts, and observed changes; avoid theory-laden shorthand when observation would be more accurate.
AI annotation and training	Neurotypical relational expectations are encoded into target labels and training corpora.	Co-design schemas with lived-experience input; separate behavior from interpretation; test performance across communication styles and proximity preferences.
Deployment and governance	Accuracy metrics obscure construct bias and epistemic harm.	Audit for differential error, contestability, and whose norms define desirable model responses; include neurodivergent expertise when reviewing relational constructs.

**Table 3** is a heuristic audit matrix. It is designed to slow interpretation and improve documentation, not to classify patients or replace case formulation.

The analysis is intentionally conceptual rather than exhaustive. It uses targeted literature synthesis and citation chaining rather than systematic database screening. This choice is appropriate for a construct-building paper, but it limits the strength of empirical claims. The proposed audit questions should therefore be read as theory-anchored questions for clinical reflection, training, service design, and future participatory research, not as validated scales or diagnostic instruments.

## 3 Autism-first evidence base and cautious transfer beyond autism

The analysis treats autism as the most developed empirical literature base for the current argument because autism research presently offers the richest work on relational mismatch, masking and camouflaging, healthcare access, double empathy, interpersonal distance, and neuroaffirmative critique (9–20, 27, 28). In that sense, the core evidentiary claim is autism-first: many clinical and service systems misread autistic communication and regulation when they expect engagement to appear as eye contact, spontaneous reciprocity, verbal ease, affective warmth, or visible receptivity to help.

The wider term neurodivergent is used only where the argument concerns more general questions of sensory load, pacing, communication format, social misattribution, masking pressure, and epistemic credibility. This broader use should not be read as implying that all neurodivergent profiles require the same accommodations or show the same relational patterns. Some people may need more immediacy, more stimulation, more external structure, or more interpersonal feedback rather than less. The construct therefore requires profile-specific, person-specific, and context-specific calibration.

This limitation is also relevant to the double empathy problem. Milton argued that social breakdowns involving autistic people should not be understood as the one-sided expression of autistic deficit, but as breakdowns of reciprocity and mutual intelligibility across differently configured social worlds (15). Subsequent work on neurotype matching and autistic peer-to-peer information transfer supports the importance of interactional fit (17, 32). At the same time, recent critique cautions against overextending the double empathy problem into a total explanation of all social mismatch (18). In this article, double empathy is used as an interactional stress-test logic, not as a universal theory of neurodivergent relationality.

## 4 Defining relational-proximity priors

Relational-proximity priors are implicit assumptions about how much closeness, warmth, reciprocity, affect display, responsiveness, spontaneity, eye contact, and visible acceptance of help should be present in order for a relationship to count as good, safe, empathic, collaborative, or therapeutic. The term prior is used deliberately. It signals that these assumptions often operate before explicit reasoning begins: they shape what clinicians notice, how teams annotate behavior, what institutions reward, and which cases feel easy or difficult to help.

The point is not that all proximity is bad or that all distance is healthy. The point is that proximity is usually normed. In many care settings, more closeness is presumed to mean better alliance; emotional warmth is presumed to be empathic; visible gratitude is presumed to index receptivity to care; and increasing spontaneity in social reciprocity is presumed to index improvement. These assumptions may sometimes be clinically useful, but they become problematic when treated as universal markers rather than context-bound hypotheses. In neuroaffirmative terms, the issue is goodness-of-fit rather than one-way correction (13–20).

A relational-proximity prior is treated here as problematically activated when three conditions occur together: first, an observed behavior is prematurely interpreted through majority-coded norms of closeness, warmth, reciprocity, gaze, pace, or gratitude; second, plausible alternative explanations such as sensory regulation, communication preference, overload, autonomy protection, stigma history, trauma response, depression, or defensive function are not seriously considered; and third, the resulting inference changes care in an escalatory, exclusionary, or documenting direction, for example by increasing pressure, pathologizing distance, labeling noncompliance, or denying access to a preferred communication format.

Relational-proximity priors also include expectations about the moral choreography of help. In many services, a person is assumed to be appropriately helped when they welcome support in a visibly receptive way, show affective appreciation, and return recognizable signals of trust. Yet acceptance of help is culturally and neurocognitively mediated. For some people, maintaining distance, requesting time, translating spoken interaction into text, or declining affectively intense support may be the very means by which help becomes acceptable. If those modes are misread as ingratitude or opposition, the clinical error is not only interpersonal but ethical.

The construct is narrower than social norms but broader than interpersonal distance alone. It includes spatial and bodily distance, affective dosage, communication tempo, gaze expectations, and expectations of mutuality. It also links directly to epistemic injustice. If the credibility of a person’s account is discounted because their mode of relationship does not match dominant expectations of warmth, coherence, or reciprocity, the person is wronged not only clinically but also as a knower (5, 21, 31).

## 5 Clinical encounters as an epistemic stress test

Clinical encounters are the most immediate site at which relational-proximity priors become consequential. A patient may answer briefly, avoid eye contact, request written questions, prefer side-by-side contact, need long pauses, decline group formats, or show limited facial and vocal affect. These observations are real. What they mean, however, is underdetermined. They might indicate defensive withdrawal, depression, mistrust, overload, alexithymic difficulty, sensory protection, literal communication style, previous relational injury, autistic regulation, or some combination of these. A good clinical model must preserve the distinction between observation, interpretation, and intervention.

A further safeguard is to distinguish regulation, accommodation, defense, and clinically significant withdrawal. A neurodivergent strategy should not be formulated as defensive merely because it reduces proximity, intensity, eye contact, spontaneity, or verbal elaboration. It may be an accommodation that preserves sensory safety, autonomy, and usable relationship. The same surface behavior may become defensive only when, in a given moment and after adequate accommodation, it rigidly prevents access to affect, need, conflict, or collaborative reflection despite sufficient tolerance. Neuroaffirmative formulation therefore asks first what a behavior makes possible, then what it prevents, and only then whether it functions defensively (12–14, 27, 28, 33, 35, 36).

A hypothetical vignette illustrates the distinction. An autistic adult in an intake interview gives brief answers, looks at the floor, asks for questions to be written down, and declines a second clinician joining the room. A proximity-based reading might call this guarded, unmotivated, poorly allied, or rejecting. A neuroaffirmative formulation first separates observations from interpretation: brief answers, gaze reduction, written-format request, and limit on room occupancy. It then tests alternative meanings: sensory load, language processing, anxiety, previous invalidation, need for predictability, depressive withdrawal, or defensive avoidance. A low-risk first intervention would be to reduce social demand, provide an agenda in writing, confirm the person’s preferred communication mode, and review meaning over time. If, after such accommodation, the person remains unable to discuss any relevant need, risk, or conflict despite reduced overload, defensive or depressive withdrawal may become more plausible. The clinical task is not to protect distance from all interpretation; it is to avoid interpreting distance before adaptation has been offered.

Psychotherapy process language is especially vulnerable to this problem because it often uses constructs that are relationally dense but operationally fuzzy. Terms such as alliance, openness, insight, motivation, cooperation, or capacity for relatedness can drift from careful clinical hypotheses into tacit reward structures. A patient who becomes easier for the clinician to read may be treated as improved even if the change occurred through masking, appeasement, or exhaustion. Conversely, a patient who becomes more explicit about needing distance, limits, or sensory protection may appear less engaged precisely at the moment they are becoming more agentic and more real within treatment.

## 6 Empathy, countertransference, rescue dynamics, and relational safety

Jon Frederickson’s interpersonal account of empathy offers a useful bridge between neuroaffirmative care and psychotherapeutic technique. It can also be read alongside an interpersonal psychiatric tradition that understands clinical meaning through human relationship rather than isolated traits (34). In a concise yet clinically rich formulation, Frederickson argues that many clinicians mistake empathy for warmth. From an interpersonal perspective, the empathic question is not how warm the therapist is being, but how this patient will experience the therapist and the intervention (33). Warmth can be soothing for one patient and invasive for another; questions can feel intrusive to one person and liberating to another. Empathy, on this view, is calibrated responsiveness, not maximal emotional closeness.

This distinction is directly relevant to neurodiversity-affirming psychotherapy. Frederickson describes adjusting whether questions or statements are used, reducing eye contact by working side-by-side, and supporting a frightened patient’s need for physical distance until feeling and reflection again become tolerable (33). These are not anti-relational maneuvers. They are relationally precise maneuvers. They treat distance, pacing, and the form of contact as part of the therapeutic intervention itself. Later work on co-creating safety and clinical thinking generalizes this stance by emphasizing tolerance, bias, communicative fit, and the patient’s moment-to-moment capacity to use what is offered (35, 36).

From a psychodynamic angle, helping roles are never psychologically empty. Professionals often feel effective, confirmed, and morally grounded when patients respond with visible warmth, gratitude, or rapid engagement. Kohut’s language of selfobject needs remains useful here, not as an accusation but as a way to recognize that helping can contain wishes to feel mirrored, useful, and relationally successful (37). When autistic or otherwise neurodivergent communication frustrates these wishes because the patient remains distant, literal, minimally expressive, or slow to reciprocate, the clinician may feel unexpectedly rejected, helpless, or compelled to intensify care.

That intensification can generate rescue dynamics and countertransference distortions. The clinician or team may increase explanatory pressure, offer more emotional intensity than the patient can bear, interpret distance as resistance, or equate low expressivity with lack of alliance. These reactions should be understood as clinically significant rather than morally blameworthy. Frustration itself becomes data: it may indicate that a hidden relational-proximity prior has been activated and that the model of good care guiding the encounter is too narrow.

## 7 Institutional sedimentation and documentation

What appears in the therapy room as a micro-mismatch often exists institutionally before the patient arrives. Many health care systems demand telephone access, spontaneous verbal self-presentation, tolerance of waiting rooms, rapid changes of plan, and repeated disclosure to unfamiliar professionals. Even when these routines are not intentionally exclusionary, they disproportionately privilege people whose regulatory style matches majority expectations around communication and proximity (9–12).

Examples are easy to find once the construct is named: mandatory telephone triage for people who communicate better in writing; bright and noisy waiting rooms; intake interviews that reward rapid autobiographical coherence; care plans that equate group participation with progress; and documentation forms that offer many ways to mark disengagement but few ways to record sensory overload, masking, communication preference, or successful low-intensity contact. These are not neutral logistics. They are institutionalized proximity preferences.

Institutional priors also shape judgments of engagement and deservingness. Patients who enter groups comfortably, answer quickly, sustain eye contact, accept help in recognizable form, and reassure staff affectively may be read as easier to help. Patients who ask for reduced stimulation, asynchronous communication, fewer people in the room, shorter meetings, or explicit negotiation of boundaries may be read as uncooperative, avoidant, or not ready. The burden of adaptation then falls disproportionately on the neurodivergent person, who is often expected to travel further out of their comfort zone than the service is willing to travel out of its own.

Documentation is a key mechanism of sedimentation. Phrases such as guarded, flat affect, poor insight, poor alliance, or noncompliant may be clinically useful only when they remain explicitly interpretive, contextual, and revisable. When written without sensory context, communication format, masking pressure, or accommodation attempts, they harden a local interpretive hypothesis into institutional truth. A neuroaffirmative record should therefore document both the observation and the conditions under which it occurred: who was present, how much sensory load was present, which medium was offered, what the person requested, what adaptation was tried, and what changed when relational demand was reduced.

## 8 AI-supported models and the scaling of misattribution

The same relational-proximity priors that operate in clinical and institutional settings may be inherited by AI-supported models. Mental health technologies do not learn from behavior in a vacuum. They learn from documentation, annotation, diagnostic categories, ratings of alliance or engagement, and decisions about what counts as cooperation, empathy, risk, progress, or noncompliance. If those inputs are already overfitted to neurotypical expectations, then AI may stabilize, legitimize, and scale them (25, 26, 30, 38).

The following examples are illustrative governance-relevant scenarios, not reports of one validated deployed system. A triage tool might treat low verbal elaboration, delayed response, or sparse affective language as elevated risk or non-engagement without knowing whether the person is overloaded, autistic, using literal style, or safer in written form. An assistive note-writing system might reproduce older documentation phrases in which flat affect, guardedness, or limited eye contact are implicitly pathologized. A conversational agent trained on generic empathy scripts may assume that greater emotional warmth, faster reciprocity, and more affiliative language are always desirable. In each case, the model inherits a theory of successful contact.

Work on epistemic injustice in mental health records and policy shows how seemingly neutral systems can discount minority voices or convert context-dependent interpretation into durable institutional truth (21, 22, 30, 39). In AI terms, labels such as flat affect, poor alliance, limited insight, or non-engagement are not innocent descriptors. They are theory-laden compression devices. Once reused for training or decision support, they can turn a local clinical misreading into a systematic model error.

A neuroaffirmative response does not require rejecting AI in mental health. It requires making relational priors explicit, contestable, and revisable. At minimum, annotation schemas should distinguish observation from interpretation; model development should include neurodivergent and lived-experience perspectives; and governance should evaluate whether systems disproportionately penalize non-normative communication, distance regulation, literal style, asynchronous communication, or low-affect expression. Human-in-the-loop governance should include neurodivergent expertise when target variables depend on relational norms.

## 9 Relational-proximity audit and implications

**Table 3** proposes a relational-proximity audit spanning clinical, institutional, documentation, and AI-supported models. Its purpose is not to replace clinical judgment with a checklist. Rather, it slows judgment at points where unaudited proximity priors are most likely to masquerade as clinical validity. The audit asks what is being observed, what is being inferred, whose comfort is being centered, what adaptation burden is being placed on the patient, and whether service or model design has already defined neurotypical relational style as the default form of cooperation.

The most immediate implication is for clinician training and supervision. Professionals should be trained to treat distance as data rather than defiance, and to ask routinely which form and dosage of contact a person can currently bear and use. Supervision should include explicit reflection on the clinician’s own relational-proximity priors: What did I hope the patient would do so that I could feel helpful? What did I interpret as rejection that might instead have been regulation? Which behaviors felt alliance-enhancing only because they matched my own comfort zone?

At service level, neuroaffirmative practice requires more than goodwill. It requires design choices: multiple communication channels, options for written or asynchronous contact, reduced sensory burden, predictable routines, transparent consent and boundary-setting, flexible pacing, and serious respect for self-determination. These are not minor customer-service preferences. They are public mental health interventions because they reduce barriers to access, lower minority stress, and broaden the range of people for whom help is practically reachable (9, 12, 29).

## 10 Safeguards, limitations, and non-goals

Several safeguards are necessary. First, the aim is not to invert the deficit model by idealizing neurodivergence or pathologizing neurotypical relationality. Neurotypical and neurodivergent perspectives should be treated as partial and complementary rather than as morally ranked worldviews. The present argument is about overfitting to majority norms, not about replacing one essentialism with another (1, 4, 5, 18, 24).

Second, the paper does not claim that distance is always better than closeness, nor that all low-affect or low-reciprocity presentations are benign. Patients can be psychotically withdrawn, severely depressed, dissociated, terrified, avoidant, or overwhelmed. Neuroaffirmative practice does not abolish diagnosis or clinical concern. It asks clinicians to slow interpretation, widen the range of hypotheses, and calibrate intervention to actual tolerance and meaning rather than to default proximity ideals (33, 35, 36).

Third, the argument does not imply that neurodivergence is a defense. Neurodivergence refers to developmental and neurocognitive variation; defense is a functional interpretation of how a behavior operates in a particular moment. Neurodivergent behaviors may sometimes be recruited defensively, but they may also be accommodations, communication preferences, sensory regulation, executive scaffolds, or stigma-protective strategies. Defensive function should be inferred only after environmental fit, sensory load, communication format, autonomy threat, and masking pressure have been considered (13, 14, 27, 28, 33, 35, 36).

Fourth, the paper is not a substitute for participatory research. Because the current empirical base is shaped heavily by autism research and because neurodivergent groups are internally heterogeneous, future refinement of the construct should involve neurodivergent scholars, clinicians, service users, and carers directly. The value of the present Conceptual Analysis lies in making a clinically actionable and institutionally auditable problem visible, not in claiming that the problem has now been exhaustively solved (2–4, 21).

Finally, the paper does not offer empirical validation of relational-proximity priors. Future work should test whether the audit questions improve formulation accuracy, patient-rated safety, service accessibility, documentation quality, or differential error in AI-supported tools. Until such work exists, the construct should be used as a disciplined heuristic: useful for slowing premature interpretation, but not sufficient for diagnosis, risk assessment, or service eligibility decisions.

## 11 Author positionality and epistemic constraints

The author writes from the position of a physician psychotherapist and social psychiatrist working in psychosomatic and public mental health settings. This positionality informed the selection of the conceptual problem and the emphasis on communicative fit, relational safety, and epistemic injustice, but it is not treated as empirical evidence. No unpublished clinical case material, personal communications, or non-public manuscripts are used as sources in this Conceptual Analysis. Because the empirical basis is strongest in autism research, broader neurodivergent transfer claims are framed as conceptual hypotheses requiring further participatory and profile-specific study.

## 12 Conclusion

Neurodiversity becomes an epistemic stress test when it reveals where psychotherapy, health care institutions, documentation practices, and AI-supported models have silently overfitted to one narrow image of good relationship: close, warm, visibly reciprocal, and socially legible in neurotypical ways. The concept of relational-proximity priors names that overfitting and helps explain why autistic and other neurodivergent people may be misread even in settings that sincerely intend to help.

A neuroaffirmative future for Public Mental Health depends on more than adding accommodations after the fact. It depends on mutual model revision. Care improves when professionals stop asking only how neurodivergent people can move toward neurotypical proximity and start asking how clinical, institutional, and computational systems can move toward forms of distance, structure, explicitness, and low-intensity reciprocity within which people can remain safe, agentic, and genuinely in relationship.
